# Supplementary material for: Inhibition of striatonigral autophagy as a link between cannabinoid intoxication and impairment of motor coordination
Source: eLife. 2020 Aug 10;9:e56811. doi: 10.7554/eLife.56811 (PMC7417168; doi:10.7554/eLife.56811)
Supplement: Transparent reporting form [file elife-56811-transrepform.docx]

***eLife’s* transparent reporting form**

We encourage authors to provide detailed information *within their submission* to facilitate the interpretation and replication of experiments. Authors can upload supporting documentation to indicate the use of appropriate reporting guidelines for health-related research (see [EQUATOR Network](http://www.equator-network.org/%20)), life science research (see the [BioSharing Information Resource](https://biosharing.org/)), or the [ARRIVE guidelines](http://www.plosbiology.org/article/info:doi/10.1371/journal.pbio.1000412) for reporting work involving animal research. Where applicable, authors should refer to any relevant reporting standards documents in this form.

If you have any questions, please consult our Journal Policies and/or contact us: [editorial@elifesciences.org](mailto:editorial@elifesciences.org).

**Sample-size estimation**

- You should state whether an appropriate sample size was computed when the study was being designed
- You should state the statistical method of sample size computation and any required assumptions
- If no explicit power analysis was used, you should describe how you decided what sample (replicate) size (number) to use

Please outline where this information can be found within the submission (e.g., sections or figure legends), or explain why this information doesn’t apply to your submission:

The sample size for each experiment was estimated on the basis of previous studies conducted by our laboratories using similar cell culture, protein marker and motor behavior approaches (*e.g.*, Bellocchio *et al.* J Neurosci 2016; Blázquez *et al.* Brain 2011; Blázquez *et al.* Cell Death Differ 2015; Chiarlone *et al.* PNAS 2014; Ruiz-Calvo *et al.* Cereb Cortex 2018). Subsequent power analysis was conducted for each parameter by using IBM SPSS software (IBM France, Bois-Colombes, France), as mentioned in the Statistics subsection of the Materials and Methods section and in the Key Resources Table. This information can be found in the respective source data files.

**Replicates**

- You should report how often each experiment was performed
- You should include a definition of biological versus technical replication
- The data obtained should be provided and sufficient information should be provided to indicate the number of independent biological and/or technical replicates
- If you encountered any outliers, you should describe how these were handled
- Criteria for exclusion/inclusion of data should be clearly stated
- High-throughput sequence data should be uploaded before submission, with a private link for reviewers provided (these are available from both GEO and ArrayExpress)

Please outline where this information can be found within the submission (e.g., sections or figure legends), or explain why this information doesn’t apply to your submission:

The number of biological replicates (number of mice, number of primary neuron preparations) is provided in the corresponding figure legends. The number of technical replicates (number of incubations within each primary neuron preparation, number of sections and fields microscopically analyzed per mouse brain, number of motor coordination trials per mouse, number of motor activity trials per mouse) is specified in the respective subsections of the Materials and Methods section. The source data files include raw numerical data and details of statistical analyses. No outliers were excluded for the statistical analyses. This study does not comprise high-throughput sequence data.

**Statistical reporting**

- Statistical analysis methods should be described and justified
- Raw data should be presented in figures whenever informative to do so (typically when N per group is less than 10)
- For each experiment, you should identify the statistical tests used, exact values of N, definitions of center, methods of multiple test correction, and dispersion and precision measures (e.g., mean, median, SD, SEM, confidence intervals; and, for the major substantive results, a measure of effect size (e.g., Pearson's r, Cohen's d)
- Report exact p-values wherever possible alongside the summary statistics and 95% confidence intervals. These should be reported for all key questions and not only when the p-value is less than 0.05.

Details of the statistical tests used can be found in the Statistics subsection of the Materials and Methods section, as well as in the corresponding figure legends and source data files. Mean, SEM and significance levels are shown, together with the individual data points (dot plots). For clarity, only *p* values lower than 0.05 were considered statistically significant, and the most relevant of them are remarked in the figures. The source data files include all raw numerical data as well as further details of statistical analyses (*e.g.*, confidence intervals, exact values of *n*, exact *p*-values, data of Tukey’s correction when appropriate, measures of power and effect size).

Please outline where this information can be found within the submission (e.g., sections or figure legends), or explain why this information doesn’t apply to your submission:

(For large datasets, or papers with a very large number of statistical tests, you may upload a single table file with tests, Ns, etc., with reference to sections in the manuscript.)

**Group allocation**

- Indicate how samples were allocated into experimental groups (in the case of clinical studies, please specify allocation to treatment method); if randomization was used, please also state if restricted randomization was applied
- Indicate if masking was used during group allocation, data collection and/or data analysis

Please outline where this information can be found within the submission (e.g., sections or figure legends), or explain why this information doesn’t apply to your submission:

Animals were assigned randomly to the different treatment groups, and all experiments were performed in a blinded manner for genotype, pharmacological treatment and viral injection, as mentioned in the Animals subsection of the Materials and Methods section.

**Additional data files (“source data”)**

- We encourage you to upload relevant additional data files, such as numerical data that are represented as a graph in a figure, or as a summary table
- Where provided, these should be in the most useful format, and they can be uploaded as “Source data” files linked to a main figure or table
- Include model definition files including the full list of parameters used
- Include code used for data analysis (e.g., R, MatLab)
- Avoid stating that data files are “available upon request”

Please indicate the figures or tables for which source data files have been provided:

Source data files are provided for all figures in a user-friendly (Excel) format. These files contain all raw numerical data as well as details of the statistical analyses, which were carried out with the GraphPad Prism 8.0 software (San Diego, CA, USA), as mentioned in the Statistics subsection of the Materials and Methods section and in the Key Resources Table.
